# Comparison of point and roadside transect methods to evaluate the abundance and richness of diurnal raptors in the arid region of Rajasthan

**DOI:** 10.1371/journal.pone.0259805

**Published:** 2021-12-08

**Authors:** Govind Tiwari, Puneet Pandey, Rahul Kaul, Hang Lee, Randeep Singh

**Affiliations:** 1 Amity Institute of Forestry and Wildlife, Amity University, Noida, Uttar Pradesh, India; 2 Conservation Genome Resource Bank for Korean Wildlife (CGRB), Research Institute for Veterinary Science and College of Veterinary Medicine, Seoul National University, Seoul, Republic of Korea; 3 Wildlife Trust of India, Noida, Uttar Pradesh, India; Zoological Survey of India, INDIA

## Abstract

Diurnal raptors show a wider distribution compared to other groups of birds including passerines, woodpeckers, and seriemas, but occur at lower-than-expected densities. Estimating the precise abundance is essential to achieve conservation goals but the methods used to estimate the populations of birds need to be appropriate to arrive at meaningful conclusions. We compared the two survey methods: roadside point count and strip transects, for estimating species richness and abundance of raptors in the arid landscape of Rajasthan. Roadside point counts and roadside strip transects were done on 50 transects between December 2019- February 2020 (with an average length of 20 km and a total distance of 3000 km) to assess the species richness and abundance of raptors. A total of 2954 observations of raptors belonging to 35 species were recorded using both methods. Mann Whitney U test result showed no significant difference in species richness and abundance estimates between both methods (p = 0.206). The point count method yielded a higher relative abundance of 2.79 individuals [10 km^2^]^-1^h^-1^ than the 1.90 individuals [10 km^2^]^-1^h^-1^ obtained during the strip transect. Also, the number of unidentified species were less for point counts. Extrapolation values indicated that both the methods do not differ much for the detection of unsampled species. The choice of survey method depends on the objectives of the study, but our results favor the use of point counts rather than strip transects to survey raptors in open habitats. The information generated from this study is expected to provide the most efficient method to study the abundance and distribution of raptors in similar landscapes.

## Introduction

Raptors are also known as birds of prey; the raptors are the top avian predators for most terrestrial ecosystems and apex predators in a given food web [1]. Raptors act as keystone species and can influence the structure of the community of which they form a part and are an indicator species—their presence serves as an index of a healthy ecosystem [2]. Higher biodiversity levels were reported at sites occupied by the raptors than at the sites from which they were absent [3]. Major threats i.e., collision with man-made objects [4], poisoning [5], the pressure of the growing human population, and pollution [6], seem to be affecting raptor populations and their distribution. Threats also include widespread deforestation and other habitat alterations; use of pesticides to maintain food production and overgrazed arid and montane regions; and the essentially unknown element of hunting and trade [7]. Possessing information on population estimates obtained by techniques that are reliable, efficient, and affordable is, therefore, key to ensuring the long-term conservation of these birds.

Raptors are rapid-moving, occur at low densities, are secretive, and range widely [8]. Such characteristics make it difficult to gather quantitative information about their abundance, population status, and distribution [9]. Survey for raptors in open habitat has commonly involved the use of two methods i.e., roadside point counts and strip transects to determine raptor distribution and abundance (both absolute density and relative abundance), but the choice of survey method depends upon the objective of the study [10]. The strip transect surveys have been conducted along roads where raptors are observed and counted from vehicles, driving at a slow speed [8, 11]. This is a common method to study raptors, affording access to most habitats, landscape features, and larger areas at a relatively low cost [10, 12]. Roadside strip transects are useful to study the raptor distribution [13], relative abundance and assemblage [14, 15], composition and diversity [16], and habitat use and preferences at a broad scale [10, 12, 17, 18]. They have been extensively used, especially in areas where information related to raptor ecology is very scarce. They may also be used for seasonal abundance and habitat usage [19], for large-scale census [20], for richness, diversity, and population monitoring [21].

Tracking some easily measurable attributes of raptor communities through strip transects can help assess large-scale habitat conservation status [22]. They can help in developing Indices of relative abundance which can be expressed as bird densities [23, 24]. The abundance indices among areas, times, and of different species can also be compared by using this method [25]. On the other hand, point count [26, 27] is amongst the most common and widely used methods to survey raptors in an open area [28]. Survey of raptors based on fixed location or at static points [27] along roadside transects at regular intervals, or in a set amount of time, has been used to estimate relative or absolute abundance [29]. It is also used in understanding bird habitat relationships and assessing the effects of landscape changes on bird populations [30–32]. Point counts done from fixed locations are very useful in comparing the results annually and between the seasons. These studies can help in maintaining a record of migrating raptors and identifying the flyways [33, 34].

Both the point count and strip transect methods are used to study raptors along with other avian communities, but both have been a subject of debate and improvement [35–39]. In estimating abundance through strip transects and point counts, one important assumption that is often violated is that birds are detected at their initial location. To overcome this bias, the snapshot approach has been suggested by Buckland et al. [40], which involves defining a moment at which the first observation of a bird is made and recording the distance from the observation point, and then monitoring the movement of the bird for remaining time so that the above-mentioned bias can be taken care. We conducted paired roadside surveys to analyze the performance of the point count and the strip transect methods in detecting raptor richness and abundance in open landscapes of Rajasthan’s arid region. The information generated from this study is expected to provide the most efficient method to study the abundance and distribution of raptors.

## Materials and methods

### Study area

**Fig 1:**
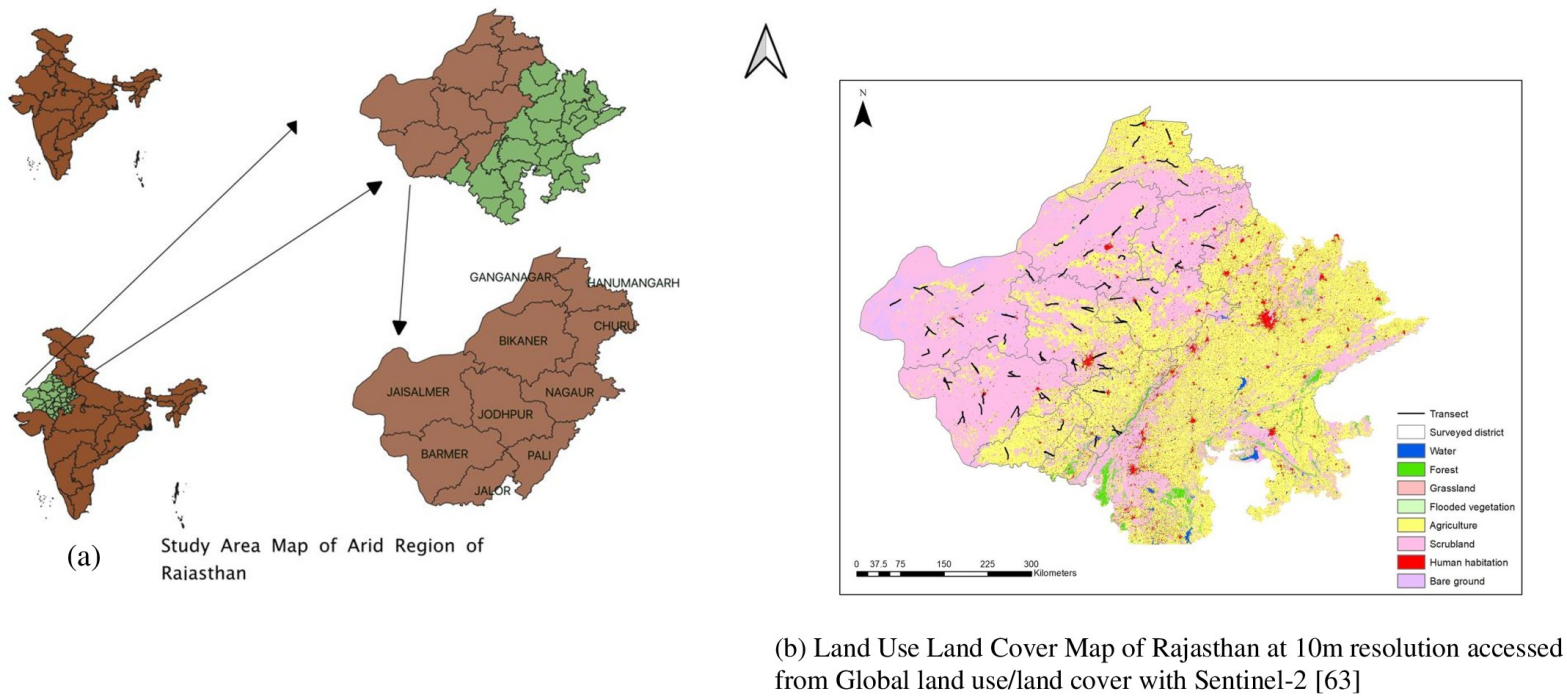
Location map of (a) study area and (b) sampling location in arid region Rajasthan from December 2019 to February 2020.

We studied raptors in the hot arid region of Rajasthan, India (Fig 1). The study area lies between 24°31′ to 30°12′ north latitudes and 69°15′ to 76°42′ east longitudes. The total area of the hot arid region covers 0.198 million km^2^ dominated by the sandy Thar desert. The region is characterized by low and erratic rainfall with an average annual rainfall of < 500 mm falling mostly (97%) in the monsoon season [41]. Temperatures vary from ≤ 0°C in winters to ≥ 50°C in summers. The area is slightly undulating with deposits of sand by inland drainage and streams with salt lakes [42]. The region has limited water resources and arable lands, high evapotranspiration, sparse vegetation, rodent infestation, absence of perennial rivers, and a sparse and nomadic human population dependent on animal rearing [43]. The rolling arid landscape is covered with northern tropical thorn forests (Champion and Seth Classification 6B). The vegetation comprises *Calligonum polygonoidis*, *Prosopis cineraria*, *Prosopis juliflora*, *Acacia capparis*, *Acacia Senegal*, *Acacia catechu*, *Anogeissus pendula*, *Butea monosperma*, and *Azadirachta indica*. Major fauna of the area is *Vulpes bengalensis*, *Gazella bennettii*, *Antilope cervicapra* and *Felis lybica ornate*. The landscape is threatened by anthropogenic activities: 22.5 million people live in the landscape with a density of around 84 persons per km^2^, which makes it the most populous desert in the world [43]. Residents are mostly (70%) engaged in agriculture, livestock farming, and mining activities [43].

#### Field survey and data collection

We conducted road surveys using a closed top car [11, 44] and carried out 50 strip transects (average length 20 km and total distance covered 3000 km) which covered the arid region of Rajasthan (Fig 1). We used 1:50,000 scale maps to select the road transects in the landscape. Transects were randomly laid based on the fisibility of the road survey. All spatial analyses including transect selection were performed using QGIS v3.18 vector tools (Quantum GIS Development Team 2021) [45]. Each road transect was surveyed using two methods: roadside point counts and strip transect. Each transect was surveyed once per month during the period from December 2019 to February 2020. A total of 300 raptor surveys were done with 50 transects surveyed once per month by two methods. For point counts, five sample points were placed on a 20 km route with each sample point 4 km apart. Every single point surveyed lasted for thirty minutes and all raptor species observed in a 1-km radius were recorded (single point sampled area of 3.14 km^2^ and total sampled area of 15.7 km^2^, with a total effort/route of 2.5 hr.).

In the strip transect, the survey was carried out with the vehicle driven at a speed of 10–30 km/hr. Raptors seen or heard at the distance of 1 km on either side of the road were recorded. Only a few times, a stop was made to confirm the identification of the raptors, and during this time, no other raptors were recorded. The mean sampled area for strip transect was 40 km^2^ (mean time:1 hour, range: 45 min-75 min). Individuals just crossing the area for a shorter time were not recorded in both methods. A method was selected for the survey at random and after its completion, the same transect is surveyed by the second method. After that, the survey was continued for the next nearby transect. We measured distances of several prominent landmarks (i.e., tree, building, electric poles, and mobile towers) with a laser rangefinder (Hawke 900m), binoculars (Nikon 10 × 52) to approximate the sample area (1 km radius), to prevent the counting of raptors outside the area. We observed in a predetermined fixed direction and recorded the number of raptors sighted in a 180° field of view. Only the birds perching, hunting, soaring, or foraging within a fixed direction were recorded. Road surveys were done from sunrise (7:00 AM) to sunset (5:30 PM), and to avoid double-counting; the behavior, color, gender, morph, and size of raptors were noted. Habitat description where the presence of a raptor was observed was also considered.

We surveyed road transects in the winter season (December 2019 to February 2020) only to avoid bias in the probability of detecting raptors due to extreme temperatures, patchy prey availability, and low availability of water. The time of the study also coincided with the time when many migratory raptors could be expected in the region. Each road transect was surveyed only on open and clear sunny days with wind speeds of <20 km/h. Surveys were avoided during foggy and rainy days. Binocular and a spotting scope (Celestron 36 x 80) were used to identify the raptors sighted and, laser range finder to measure distance when required. Nikon D500 camera with 150–600 mm lens was used to take photographs wherever photo ID was needed. Photographs were cross-checked with the description of raptor species given in (a) Birds of the Indian subcontinent [46] and (b) Birds of Prey of the Indian Sub-continent [47] to confirm the identity of the species. A closed roof Mahindra TUV 300 vehicle was used to carry out road surveys.

### Data analysis

**Fig 2:**
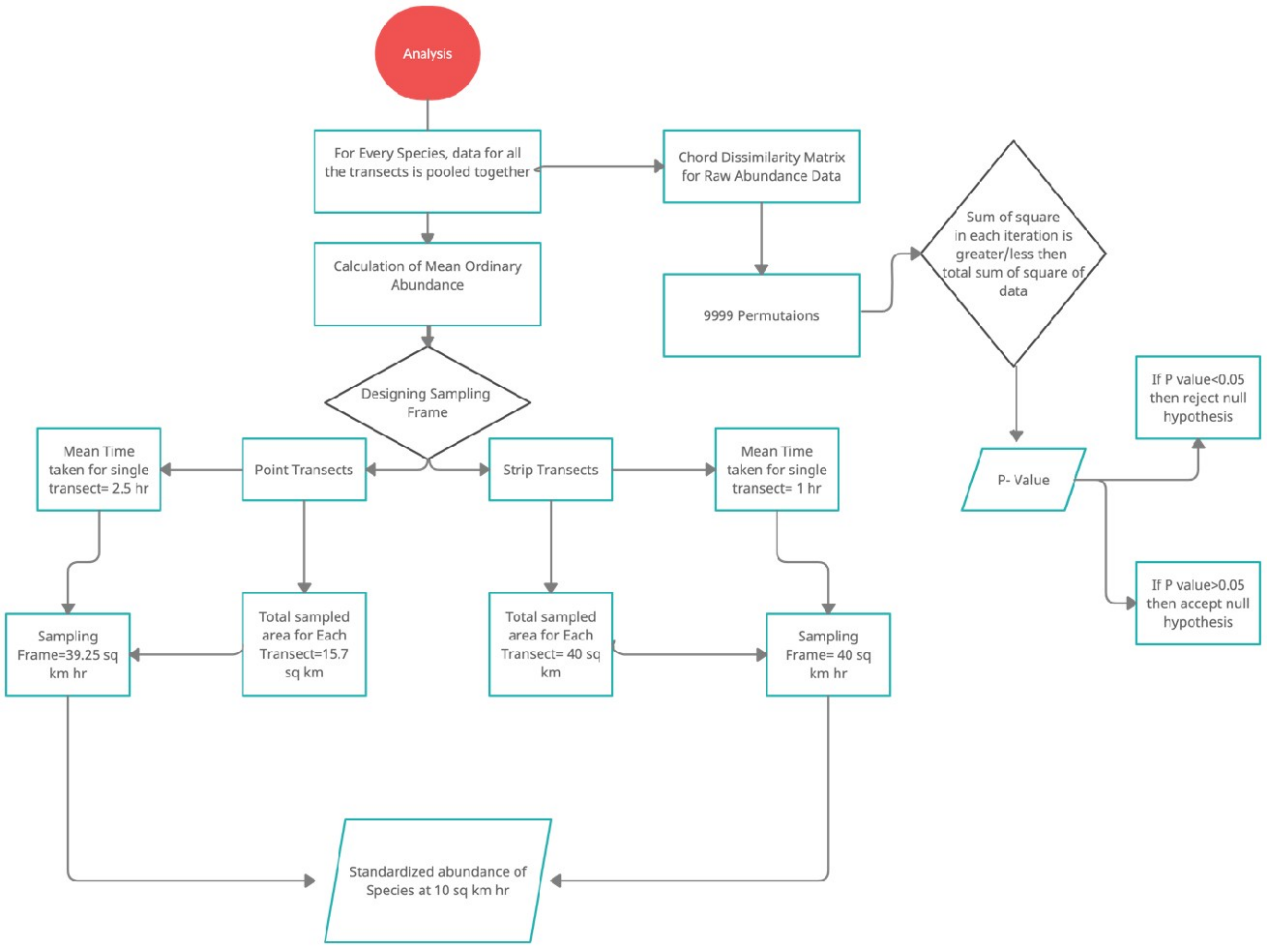
Flow chart for standardized and raw abundance analysis of raptors in winter seasons from roadside point count and strip transect methods in the arid region of Rajasthan.

Abundance data of all transects for each species were pooled together to estimate raw abundance (S1 Data). Standardized abundance was obtained from both methods for each species (Fig 2). Analysis of variance, based on randomization (permutation) tests were used to compare species abundance between the point count and strip transect methods. Chord dissimilarity matrix to species abundance data was made and 9999 permutations were run to calculate the sum of squares and probability p (Qb_O≥_Qb_A_). The algorithm involves a sum of squares test (Q_b_) which is based on dissimilarity between groups [48, 49] and the algorithm is implemented in MULTIV software and was used for the analyses. Here probability p is defined as the ratio of the sum of squares calculated in each iteration, that is greater than or equal to the sum of squares calculated for the sample. Test between methods was performed as a block design, with permutations restricted to each sample. All the analyses were done according to the methodology suggested by Zilio et al. [15, 50]. Data are presented as mean ± standard deviation.

The species richness and rarefaction curves were plotted in R (Version 1.4.1717) by using the iNEXT package [51]. iNEXT function was configured at 40 knots and 200 bootstraps replications with 95% confidence intervals. Mann Whitney U test was used to compare the richness from both methods. Diversities were compared at the same sample coverage allowing for a standardized comparison of raptor assemblage between methods. Abundance data for both methods was used as an input to make graphs for different hill numbers which represented Species Richness (q = 0), Shannon Diversity (q = 1), and Simpson Diversity (q = 2) [52, 53]. Standardized plots using the ggplot2 function in package R were made which represented sample coverage for both rarefied and extrapolated data. Species were placed into subfamilies according to the classification suggested by [54], and species richness for both the methods was compared for each subfamily by making boxplots using the ggplot2 package in R. Data for species richness for 50 transect points was pooled and plotted as jitter points in the graph. All the analysis were conducted in R [55] and figures were produced using the package ggplot2 [56].

## Results

**Fig 3:**
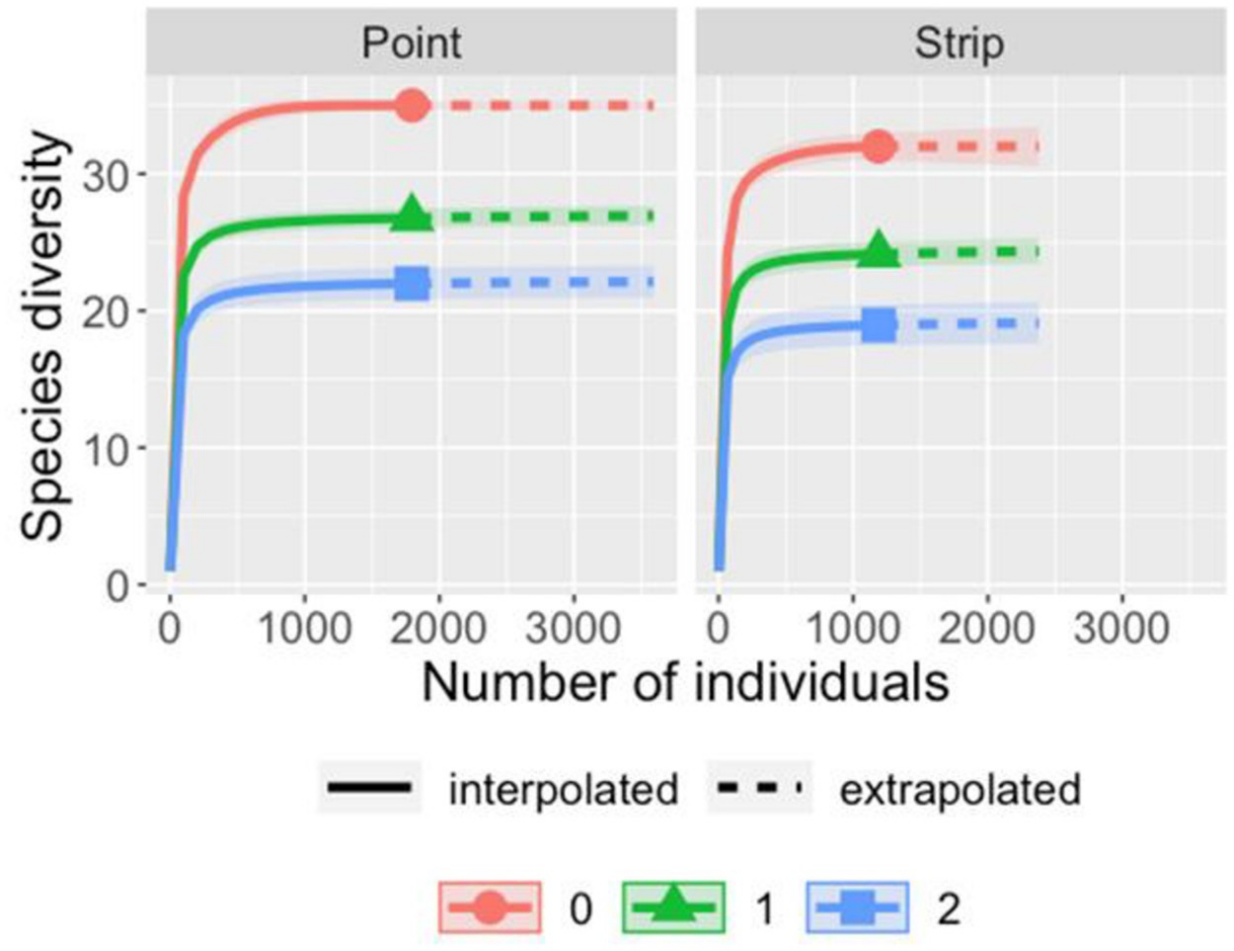
Species rarefaction plot for three hill numbers; q = 0 (Specie Richness); q = 1 (Shannon diversity); q = 2 (Simpson diversity) in the arid region of Rajasthan.

**Fig 4:**
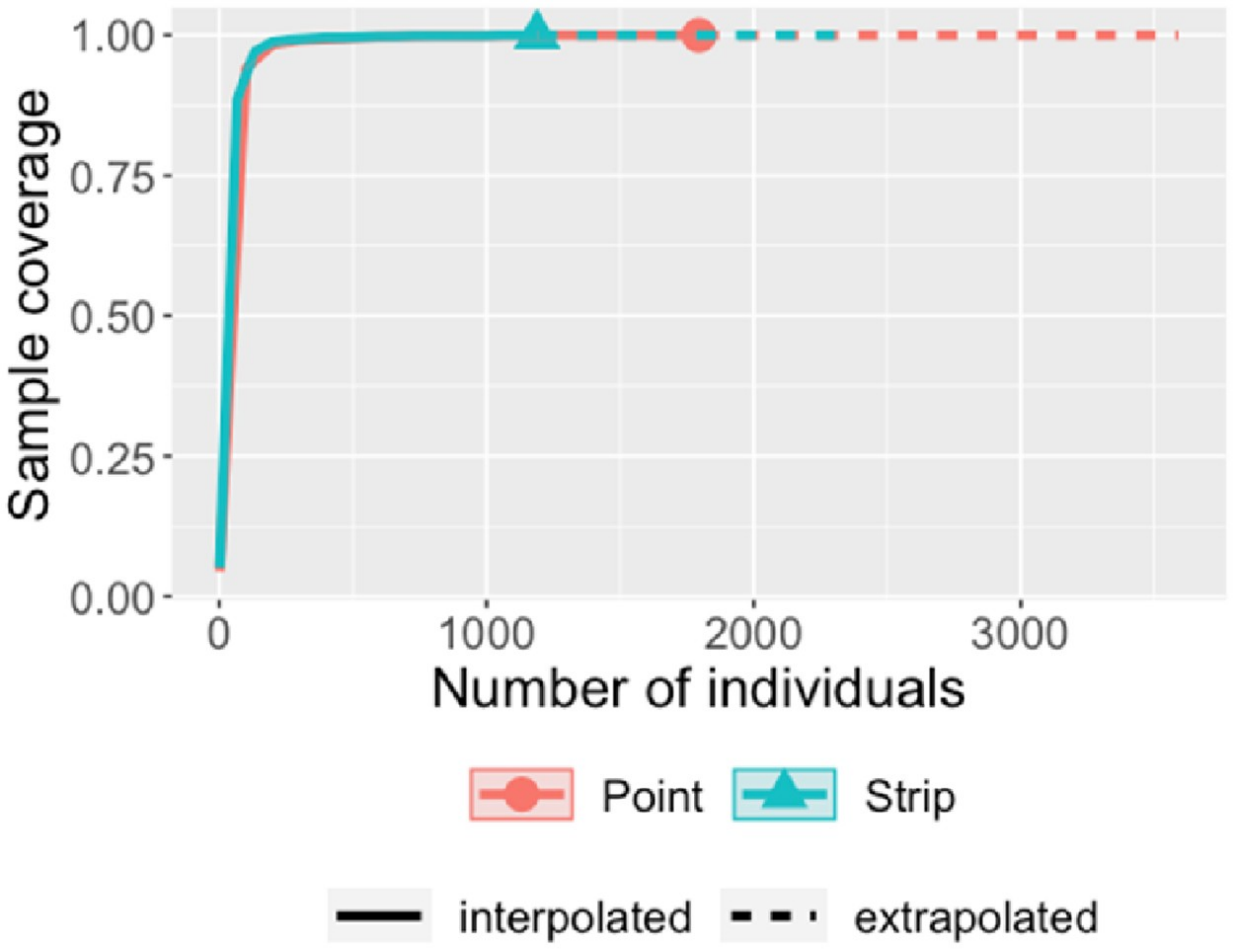
Sample coverage concerning the number of individuals of l raptors in winter seasons using roadside point count and strip transect methods in the arid region of Rajasthan.

**Fig 5:**
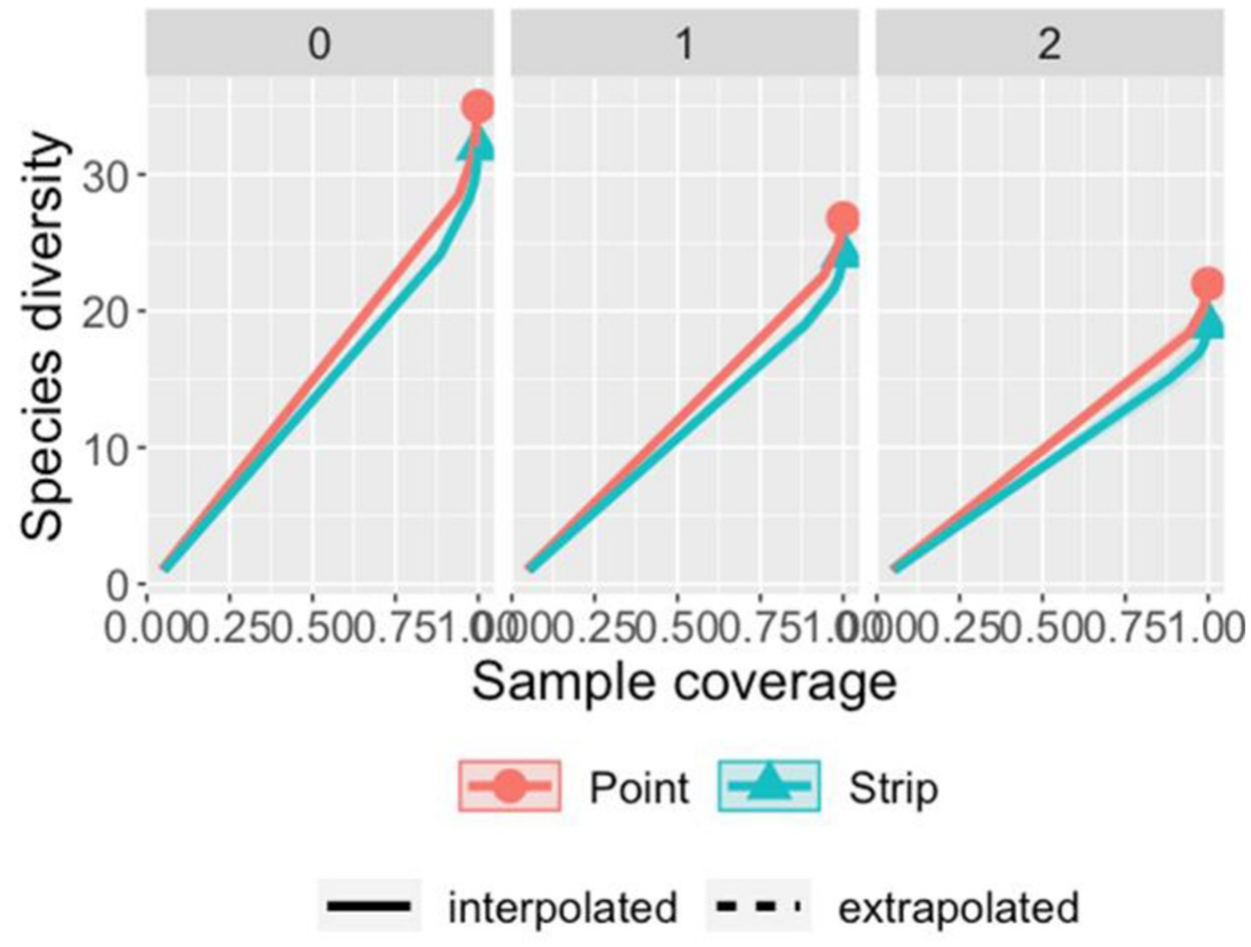
Sample coverage concerning species diversity of raptors in winter seasons using roadside point count and strip transect methods in the arid region of Rajasthan.

**Fig 6:**
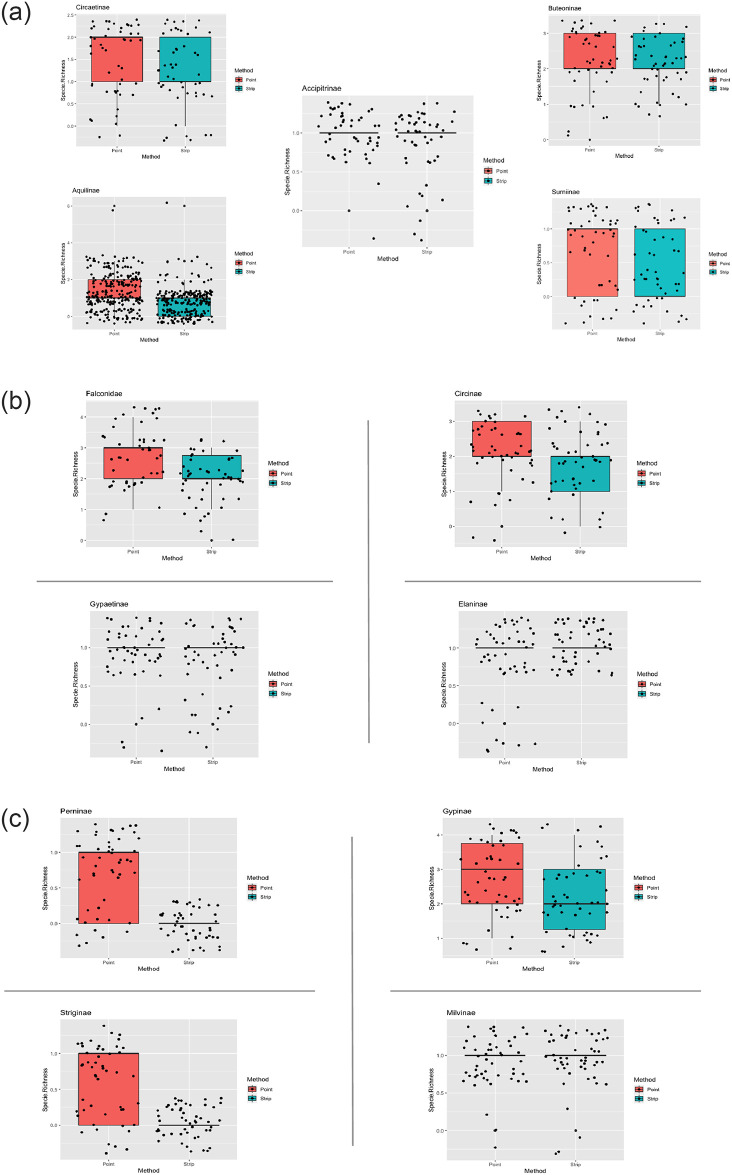
Boxplots with jitter points representing the species richness for 13 different sub-families and 1 species unidentified of raptors in winter seasons using roadside point count and strip transect methods in the arid region of Rajasthan.

A total of 35 raptor species were detected during the sampling period. The number of observations were very few for seven species, hence only 28 species were considered for species abundance estimation. A total of 2954 observations of raptors were made, involving 35 species and 14 subfamilies (Table 1). Point counts produced more observations (1777) as compared to strip (1177) (Table 1). The species richness estimated from different estimators shows that the point counts recorded slightly higher richness (35) than that estimated from strip transects (32) (Table 2). Mann Whitney U test result showed no significant difference from both the methods (p = 0.206). The rarefaction plot also showed similar estimates from both methods (Fig 3). Total species detected by sample and coverage-based rarefaction were more for point count. Confidence interval (95%) converged for both methods during extrapolation which shows that strip transects and point counts do not differ significantly in the detection of unsampled species, and overlapping intervals between two methods with low-level of sampling indicates that there was no significant difference between species richness by both the methods. A similar sample completeness curve for both methods was obtained as a function of the number of individuals (Fig 4). Both the curves plateaued at the same sample point in the interpolated region which suggests that both methods reached detection limits concerning species present in the study region and their respective abundance (Fig 5). Point counts appeared better for detecting species of *Aquila* eagles and harriers, whereas both methods gave similar richness for sub-family Buteoninae and Circaetinae. The number of species that went unidentified was more with strip transects. Box plots with jitter points were similar for Accipitrinae and Gypaetinae, whereas species richness obtained from both methods were similar, but the dispersion of jitter points shows that species were more observed more during point transects. Elaninae’s dispersion of jitter points shows that it was more frequently observed during strip transects. Both Striginae and Perninae were observed only during point transect (Fig 6). Higher species abundance was estimated for the point count method (*p* = 0.001). Abundance recorded with point counts was 2.79 individuals [10 km^2^]^-1^h^-1^, while with strip transects it was 1.90 individuals [10 km^2^]^-1^h^-1^. Species that prefer foliage like the oriental honey buzzard and the changeable hawk-eagle, and that prefer stone quarries such as the rock eagle-owl were seen only during point counts. Abundance estimates for cinereous vulture and white-rumped vulture were almost similar in both methods.

**Table 1:** Estimated abundance of raptors from roadside point count and strip transect methods in the arid region of Rajasthan.

Sub-family	Species	Point count			Strip transect		
		Total observation	Raw	Standardized	Total observation	Raw	Standardized
Accipitrinae	Shikra (*Accipiter badius*)	125	0.83±0.42	0.21±0.10	53	0.35±0.23	0.08±0.05
Aquilinae	Eastern imperial eagle (*Aquila heliaca*)	74	0.49±0.42	0.12±0.10	42	0.27±0.26	0.06±0.06
	Bonelli’s eagle (*Aquila fasciata*)	46	0.30±0.27	0.07±0.07	29	0.19±0.16	0.04±0.04
	Booted eagle (*Aquila pennata*)	49	0.32±0.26	0.08±0.06	30	0.19±0.16	0.04±0.04
	Steppe eagle (*Aquila nipalensis*)	72	0.47±0.32	0.12±0.08	44	0.29±0.18	0.07±0.08
	Tawny eagle (*Aquila rapax*)	89	0.59±0.29	0.15±0.07	49	0.32±0.24	0.08±0.06
	Indian Spotted Eagle* (*Clanga hastata*)	2	-	-	2	-	-
	Greater Spotted Eagle* (*Clanga clanga*)	3	-	-	1	-	-
	Changeable Hawk Eagle* (*Nisaetus cirrhatus*)	1	-	-	1	-	-
Buteoninae	Common buzzard (*Buteo buteo*)	57	0.37±0.31	0.09±0.08	36	0.23±0.28	0.05±0.07
	Long legged buzzard (*Buteo rufinus*)	77	0.51±0.31	0.13±0.07	40	0.26±0.16	0.06±0.04
	White eyed buzzard (*Butastur teesa*)	66	0.43±0.30	0.12±0.07	74	0.49±0.33	0.12±0.08
Circaetinae	Crested serpent eagle (*Spilornis cheela*)	38	0.25±0.21	0.06±0.05	26	0.17±0.17	0.04±0.04
	Short toed snake eagle (*Circaetus gallicus*)	50	0.33±0.24	0.08±0.06	39	0.25±0.19	0.06±0.04
Circinae	Marsh harrier (*Circus aeruginosus*)	67	0.44±0.36	0.11±0.09	49	0.32±0.20	0.08±0.07
	Montagu’s harrier (*Circus pygargus*)	47	0.31±0.26	0.07±0.06	31	0.20±0.18	0.05±0.04
	Pallid harrier (*Circus macrourus*)	41	0.27±0.23	0.06±0.06	27	0.17±0.17	0.04±0.04
Elaninae	Black wing kite (*Elanus caeruleus*)	106	0.70±0.50	0.17±0.12	110	0.73±0.31	0.18±0.79
Falconinae	Common kestrel (*Falco tinnunculus*)	49	0.32±0.24	0.08±0.06	50	0.33±0.16	0.08±0.08
	Laggar falcon (*Falco jugger*)	49	0.32±0.31	0.08±0.08	30	0.23±0.20	0.04±0.05
	Peregrine falcon (*Falco peregrinus*)	37	0.24±0.20	0.06±0.05	18	0.11±0.16	0.02±0.04
	Red necked falcon (*Falco chicquera*)	45	0.29±0.25	0.07±0.06	20	0.13±0.17	0.03±0.01
	Saker Falcon* (*Falco cherrug*)	2	-	-	1	-	-
	Merlin* (*Falco columbarius*)	2	-	-	1	-	-
	Hobby* (*Falco severus*)	3	-	-	2	-	-
Gypaetinae	Egyptian vulture (*Neophron percnopterus*)	79	0.52±0.32	0.13±0.08	47	0.31±0.24	0.07±0.06
Gypinae	Cinereous vulture (*Aegypius monachus*)	38	0.25±0.21	0.06±0.05	32	0.21±0.17	0.05±0.04
	Eurasian griffon (*Gyps fulvus*)	43	0.28±0.18	0.07±0.04	29	0.19±0.18	0.04±0.04
	Indian vulture (*Gyps indicus*)	39	0.25±0.22	0.06±0.07	24	0.15±0.17	0.03±0.04
	White rumped vulture (*Gyps bengalensis*)	37	0.24±0.21	0.06±0.05	33	0.21±0.19	0.05±0.04
Milvinae	Black kite (*Milvus migrans*)	212	1.41±0.83	0.36±0.21	162	1.08±0.47	0.27±0.11
Perninae	Oriental honey buzzard (*Pernis ptilorhynchus*)	42	0.27±0.23	0.07±0.06	-	-	-
Pandioninae	Osprey* (*Pandion haliaetus*)	2	-	-	1	-	-
Striginae	Indian eagle owl (*Bubo bengalensis*)	36	0.23±0.22	0.06±0.05	-	-	-
Surniinae	Spotted owlet (*Athene brama*)	43	0.28±0.24	0.07±0.06	24	0.15±0.17	0.03±0.05
	Species Unidentified	9	0.05±0.12	0.01±0.03	20	0.13±0.16	0.03±0.04
	Overall	1777	11.61±2.01	2.79±0.76	1177	7.62±0.51	1.90±0.15

Standardized Abundance: Individuals. [10 km^2^h^-1^]

**Table 2:** Estimates of species richness of raptors in winter seasons from roadside point count and strip transect methods in the arid region of Rajasthan.

Method	Diversity	Observed	Estimated	S.E.	UCL	LCL
Point count	Species richness	35.00	35.00	0.19	35.40	32.20
	Shannon diversity	26.77	27.03	0.40	27.83	24.52
	Simpson diversity	21.98	22.24	0.69	23.60	20.90
Strip transect	Species richness	32.00	32.00	0.49	33.26	30.02
	Shannon diversity	24.13	24.45	0.52	25.49	22.61
	Simpson diversity	18.95	19.24	0.80	20.82	17.79

## Discussion

Species richness and abundance are commonly used to generate complex diversity indices that are dependent on the quality of these estimates [57]. Integrated curves based on sampling theory that smoothly link rarefaction (interpolation) and prediction (extrapolation), standardize samples based on sample size or sample completeness, and facilitate the comparison of biodiversity data [56]. Our results show that in the rarefaction plot 95% confidence interval of three curves intersected significantly and the order for diversity was Species Richness > Shannon Diversity > Simpson Diversity. The confidence interval of all three overlapped which shows that there is no significant difference between the expected diversity indices. Curves for all three hill numbers for both methods increase steeply concerning the number of individuals and plateaued during extrapolation. Sample coverage for both methods showed a similar estimation for the coverage deficit, which suggests the probability that new, previously unsampled species can be found in the sample if it is enlarged by one individual [57]. Sample coverage did not vary much between the observed point and endpoint for both the methods which again shows that both methods have reached their limit concerning sample coverage. Rarefaction and Extrapolation curves of sample coverage (Fig 4) showed that sample coverage was more than 90% during both the methods so it can be inferred that the highest coverage value did not differ from base coverage. Simpson index (q = 1) and Shannon diversity (q = 2) were higher for point counts (Fig 3).

The activity pattern of the raptor also varied throughout the day. Black kite was the most seen raptor (374 observations) followed by the black-winged kite (216 observations), shikra (178 observations), tawny eagle (138 observations), white-eyed buzzard (140 observations), and Egyptian vulture (126 observations). Red-headed vulture (*Sarcogyps calvus*) was seen only once during the study period and observations of saker falcon (*Falco cherrug*), Eurasian hobby (*Falco Subbuteo*), merlin (*Falco columbarius*), greater spotted eagle (*Clanga clanga*), Indian spotted eagle (*Clanga hastata*), osprey (*Pandion haliaetus*), and changeable hawk-eagle (*Nisaetus cirrhatus*) were very few and hence they were not used in the analyses. The detectability of birds may vary because of species, habitat, observer, and many other factors [39]. Our results showed that point counts returned a higher abundance as compared to strip transects whereas a study done by [58] in a forest area shows that strip transects were more time-efficient, and more species and higher richness were obtained as compared to point count. However, in our study, it was seen that two species that prefer foliage and are inconspicuous: crested serpent eagle and spotted owlet could be sighted more frequently on point counts as compared to strip transects. The crested serpent eagle was observed mostly in the foliage along with the Indira Gandhi Nahar project canal network and around the wetlands of the Bikaner region. Red-headed falcon prefers the open habitat, interspersed with groves of trees, cultivation, reservoirs, and villages. It is widely distributed but sightings are difficult due to its inconspicuous nature [59]. This species recorded very low observations during strip transects as compared to point counts.

Detection functions for species could be different among different habitat types and can also change over the years [12]. It was found that *Buteo* species were seen more often on transects near cultivated areas (212 out of 350 observations). *Buteo* spp. were observed perching and foraging in cultivated areas, and all three species were recorded more during point counts. Booted eagle was absent in the true desert region but was observed near the Gajner lake (Bikaner) and Gadisar lake (Jaisalmer), both in strip transects and point counts. Pallid harrier is a rare, highly dispersed, and poorly studied raptor [60]. Pallid harrier prefers grasslands and was recorded more on transects in and nearby protected areas (41 out of 68 observations). Pallid harrier spends most of the day perching on the ground inside the grass cover. It is thus apparent why it was seen more on point counts. Tawny eagle, which is a resident raptor species of the region showed similar abundance to the migratory steppe eagle on strip transects. However, while on point counts, the steppe eagle was seen more than the tawny eagle, although the abundance of tawny eagle was higher. Bonelli eagle was observed nesting in rocky and hilly terrain, mostly during point counts. The efficiency of the point count method in estimating the abundance of shy birds concealed in vegetation could be related to being better able to spot them while stationary than moving [15].

Strip transects are a cost-effective and efficient way of estimating species abundance [61] and can generate suitable results for common raptor species [23, 24]. In this study, the black-winged kite was sighted more during strip transects. When compared to the total time and effort that is needed for the completion of the survey, strip transects are more effective as compared to point counts because birds can be sampled without breaks. A single strip transect took nearly an hour to complete, while the single point count took 2.5 hours. The shorter survey time is a potential advantage of this method [23, 24]. A careful approach needs to be followed while surveying raptors from strip transect as small and inconspicuous raptors might go unnoticed if the driving speed of the vehicle is not low. Common kestrel and black-winged kite were seen flying and perching near the roadside during the study period and both gave more raw estimates of abundance in strip transects.

The effectiveness of point count can be increased if a vantage point is selected in a suitable habitat beforehand by undertaking a preliminary survey, which can increase the detection probability of the target species [62]. Point count may detect more raptors as compared to strip transects but they are also time-consuming. Duration of halt at a point can be decreased [63] if the view from a single point is large enough to cover the whole area. Strip transects are more effective if the objective is to maximize the number of birds detected per survey time while point counts are effective if the objective is to maximize the number of birds and nests located in an area [64].

## Conclusion

Raptors are an important part of the ecosystem. They are affected by many environmental factors. Monitoring raptors can give us information about the change in environmental quality. Survey methods involve data collection on numbers and distribution which can be used to monitor changes over time. Raptors occur at low density, so the choice of the survey method influences their abundance estimates. In this study, we have focused on the analysis and comparison of two survey methods. Strip transects are a cost-effective method but point counts gave more precise estimates. Both point count and strip transect used for the study gave similar species richness, but results indicate that abundance obtained from point counts is more when compared to strip transects. We suggest that point count can be more useful in estimating the abundance and distribution of raptors in open habitats. Thus, this study can provide meaningful insights in deciding between point count and strip transect methods to survey raptors in a similar habitat.

## Supporting information

S1 Data (XLSX)
